# Exploratory Analysis of the Links among Food Consumption Profiles, Prenatal Androgens, and Selected Measures of Quality of Life

**DOI:** 10.3389/fpubh.2016.00240

**Published:** 2016-10-26

**Authors:** Klaudia Modlinska, Wojciech Pisula

**Affiliations:** ^1^Institute of Psychology, Polish Academy of Sciences, Warsaw, Poland

**Keywords:** diet, digit ratio, 2D:4D, prenatal sex steroids, meat consumption

## Abstract

Prenatal sex steroids play a vital role in the development of the whole organism, and therefore also the brain. Exposure of the fetus to testosterone seems to be of special importance both for typical development and pathology. The key factor impacting offspring development (including prenatal androgen levels) appears to be diet, both in terms of shortage and excessive intake of certain food products. Prenatal steroid levels are measured using the ratio of the lengths of the second and fourth fingers (2D:4D). So far, the digit ratio (2D:4D) has been shown to correlate negatively with prenatal testosterone and positively with prenatal estrogen. Numerous correlational studies found relationships between the 2D:4D phenotype and differences in magnitude of many psychological traits. Certain social and demographic variables also correlate with the digit ratio. The present paper offers a preliminary analysis of correlations between diet, prenatal hormones’ levels (established based on the digit ratio), and selected social variables. One of the findings is that countries with high meat consumption present the so-called masculine digit ratio, while countries with plant-based diets – a feminine digit ratio.

## Introduction

Since the ground-breaking work on organizational vs. activational effects of hormones (1), a significant amount of accumulated data confirm the relationship between sexual dimorphism (and differences in sexual characteristics within the same sex) and fetal exposure to prenatal sex steroids (prenatal estrogens and prenatal androgens) (2–5). Fetal testosterone level is thought to be particularly important (6, 7). Individual variations in testosterone levels tend to be associated with a number of internal and external factors. In animals producing large litters, the level of prenatal testosterone depends to a large extent on the number and position of male offspring in a given litter (8). External factors are hypothesized to be the effects on the pregnant female of a variety of substances in her environment, such as industrial by-products and compounds of e.g., plant origin (phytohormones). Some of these substances can affect the hormonal balance of the mother and the fetus (9). Multiple researchers have reported correlations between pesticides, such as DDT or DDE, and child development (10, 11). Another example is a component of commonly used plastics called biophenol A, and its effects on changes in the rate of sexual development and reproductive functions (12). Prenatal testosterone levels are also affected by maternal smoking (13). Stress and malnourishment during pregnancy can also change endogenous hormone concentration (14). Nevertheless, the key factor impacting offspring development (including the levels of prenatal androgens) appears to be diet, both in terms of shortage and excessive intake of certain products (14–21). Since diet is subjected to dynamic changes resulting from increasing availability and supply of food products, it appears to be a key environmental influence on human physiology, including fetal development. Diet is also a reflection of social status (22, 23). Higher-quality diets are consumed by better educated and more affluent groups, and lower quality diets prevail in groups of lower socioeconomic status (22). Data published by Food and Agriculture Organization of the United Nations (FAO) highlight the key role of changes in dietary profiles in economically growing countries, consisting, among others, in a significantly higher consumption of meat – see also Ref. (24). It seems that other components of diets also affect nutritional profiles of entire nations. A probable relationship between national dietary profiles and digit ratio patterns is the focus of attention in the present article.

In order to estimate fetal hormone levels in particular groups, various indicators are taken into consideration. One widely used biomarker of prenatal sex steroids’ levels is the digit ratio (25–28). The digit ratio is the relative length of the second (index) and fourth (ring) finger (2D:4D). The digit ratio has been shown to be negatively correlated with prenatal testosterone and positively with prenatal estrogen (29). Sexual dimorphism in digit ratio emerges *in utero* (30, 31). It is hypothesized that the genes that control urogenital development can also affect limb formation (32). On average, men have lower digit ratios than women (7). The relationship between the levels of prenatal sex steroids and digit ratio has been empirically validated, e.g., in studies involving hormonal manipulation (33, 34). More evidence comes from research on autism, Asperger’s syndrome, hyperactivity, etc. (35–37). Digit ratio is related to conditions where prenatal testosterone differs from normal levels or there are differences in sensitivity to testosterone. This includes CAH, which is related to high prenatal androgen and low 2D:4D (38, 39), androgen insensitivity, which is related to high 2D:4D (40) and Klinefelter’s syndrome, which is associated with low levels of prenatal testosterone and high 2D:4D (41–43). Individual differences in digit ratio are hereditary (44, 45). Although there are differences between ethnic populations, sexual dimorphism in digit ratio is universal (46, 47).

Many correlational studies have found correlations between the 2D:4D phenotype and differences in levels of multiple sex-dependant psychological traits (27, 48–50). For example, individuals with low digit ratio score high on dominance scale (49), mainly in terms of aggressive dominance (51). This confirms the hypothesis that high level of prenatal testosterone and/or low level of prenatal estrogen is associated with dominance. Relative concentration of prenatal testosterone and prenatal estrogen is thought to affect reproduction, pubertal development, strength and fitness, aggression and social behavior (7, 25, 52, 53), as well as attractiveness for the opposite sex (54).

Certain social and demographic variables also correlate with digit ratio. For example, in societies where women have lower and men higher 2D:4D than expected for sex, there is a higher representation of women in parliament and greater female workforce participation (26). This is associated with high dominance scores in women (49). This factor may influence greater accessibility of politics and job market to women. Similar results are observed in studies on women employed in professions considered to be typically masculine. These women also have a lower than expected 2D:4D (55). Furthermore, rich societies have a negative res2D:4D (2D:4D in women is similar to 2D:4D in men). It seems that greater sex equality enables more women to work, which is particularly important in the aging Western societies. This may indicate that high testosterone *in utero* is associated with the formation of characteristics, preferences, and abilities required in traditionally masculine professions.

## Materials and Methods

The aim of this paper is preliminary exploration of the relationships between diet, prenatal hormones (measured by digit ratio), and social functioning characteristics. In order to evaluate the possible effect of the main environmental factor, such as diet profile, in shaping the sexual dimorphism in the general populations across the countries, the multivariate approach has been adopted. The profile of the food consumption involves various food components, and therefore, all basic aspects of consumption should be taken into account. The first stage of the analysis involves clustering of countries into the homogeneous groups on the basis of their profile of food consumption (wheat, poultry, etc., consumption per capita per year). The second stage is a description of clusters, which is supposed to provide a general picture of how people utilize the food sources in their countries. This stage of analysis includes also the comparison of the groups of countries in terms of the sexual dimorphism measured by 2D:4D. Although this kind of analysis does not allow to formulate any casual explanations, it may provide a valuable hint for more detailed research on the role of diet in prenatal androgens level regulation.

Dietary profiles of selected countries were analyzed. For the analysis were selected countries for which there are reliable data on digit ratio in their populations (26). The analysis of dietary profiles was based on the most popular and common to all these countries food products such as meat, eggs, milk, wheat, vegetables, and fruits. Products consumed only in certain countries or eaten in small amounts have been excluded from the analysis. In order to extract general style of food consumption, a multivariate approach was adopted. The cluster analysis (*K*-means method) was administered in order to group countries presenting similar food consumption profiles.

Information on basic food products and alcohol intake was obtained from the Food and Agriculture Organization of the United Nations database.^1^ Data from the years 2000–2011 were taken into account.

Extracted in the cluster analysis groups of countries were compared in terms of the digit ratio profile, which dominates in the country’s population. Digit ratio values were sourced from Manning et al. (26). They published a chart with digit ratios of nationals of 29 countries (including non-European ones). The list presents mean digit ratios for both hands and both sexes, and residuals (res2D:4D) for either sex calculated as residuals of the regression female 2D:4D on male 2D:4D and considered to be the measure of per nation sex differences. Negative residuals indicate lower than expected 2D:4D in women compared to men, positive ones higher than expected 2D:4D in women compared to men.

The next step was to compare the extracted in the cluster analysis groups of countries in terms of demographic variables associated with the quality of life. The demographic variables previously described by Manning et al. (26) were excluding from the analysis. It seems that the quality of life level may be correlated with the nutritional profile of the country (e.g., through the relation between income and diet quality) and with its digit ratio profile (22, 26).

Demographic data were sourced from The World Factbook,^2^ databases published by the Central Intelligence Agency, covering fields such as history, people, government, economy, geography, communications, transportation, military, and transnational issues for 267 world entities. It is the most comprehensive and constantly updated database of demographic data. The database is used by a wide variety of folks including US Government officials, researchers, news organizations, corporations, geographers, teachers, professors, librarians, and students from all over the world. Information in The World Factbook is collected from a wide variety of US Government agencies, as well as from hundreds of published sources.

All data used for the analyses in this paper are compiled in additional materials.

## Results

The data described above were analyzed using the multidimensional approach. The initial step was cluster analysis conducted on selected countries.

### Stage I. Cluster Analysis

The analysis was conducted using the *K*-means method of clustering. The procedure used information on the intake of basic food products in 29 countries (26). Three clusters were identified. Cluster 1 (WSS = 27.041) is made up of 4 countries (Greece, Italy, Romania, and Turkey), cluster 2 (WSS = 67.711) of 14 countries (Austria, Belgium, Bulgaria, Croatia, Czech Republic, Denmark, France, Germany, Hungary, Netherlands, Poland, Spain, Sweden, and Switzerland), cluster 3 (WSS = 76.225) of 11 countries (Argentina, Australia, Canada, Finland, Iceland, Ireland, New Zealand, Norway, Portugal, United Kingdom, and United States).

Table 1 shows the mean individual food product consumption in each of the three identified clusters. Two products included in the analysis did not discriminate between the clusters (milk and fruits). For all the remaining products, the differences in consumption between clusters were statistically significant.

**Table 1:** **Food supply (kilograms per capita per year) in each cluster**.

		Cluster 1	Cluster 2	Cluster 3
**Bovine**	Mean (SD)	14.42 (8.92)	15.40 (7.37)	28.07 (12.98)
	Fisher’s *F*	5.613 (*p* = 0.009)		
**Pig meat**	Mean (SD)	24.68 (17.18)	41.79 (12.93)	25.95 (9.35)
	Fisher’s *F*	6.281 (*p* = 0.006)		
**Poultry**	Mean (SD)	16.04 (1.71)	19.57 (5.73)	29.07 (10.29)
	Fisher’s *F*	6.615 (*p* = 0.005)		
**Eggs**	Mean (SD)	10.89 (1.97)	13.21 (2.35)	9.59 (2.20)
	Fisher’s *F*	8.123 (*p* = 0.002)		
**Milk**	Mean (SD)	61.14 (42.54)	71.62 (31.61)	93.44 (36.20)
	Fisher’s *F*	1.783 (*p* = 0.188)		
**Wheat**	Mean (SD)	152.11 (30.12)	95.75 (14.79)	88.24 (14.21)
	Fisher’s *F*	21.631 (*p* < 0.001)		
**Fruits**	Mean (SD)	120.47 (41.44)	92.17 (28.55)	111.13 (14.71)
	Fisher’s *F*	2.603 (*p* = 0.093)		
**Vegetables**	Mean (SD)	210.22 (46.61)	102.43 (19.66)	100.49 (34.21)
	Fisher’s *F*	22.777 (*p* < 0.001)		
**Fish, seafood**	Mean (SD)	14.54 (9.76)	18.91 (11.15)	34.32 (23.19)
	Fisher’s *F*	3.380 (*p* = 0.049)		

*Mean values and SD. Fisher F for clusters comparisons*.

Based on the above data, consumption profiles of basic food products were established for each cluster. Cluster 1 is characterized by high intake of wheat and vegetables (almost two times higher than the two other clusters) and the lowest consumption of meat (all types) of all clusters. Eggs consumption is low, similar to that for cluster 3.

In cluster 2, pork (pig meat) was the most consumed type. The consumption of eggs was also the highest of all three clusters. The values for foods such as poultry, fish, bovine, and wheat were in the middle, although in the case of bovine, fish, and poultry, they approximate the low values in cluster 1, while wheat consumption is similar to the lowest value in cluster 3.

Cluster 3 features a particularly high consumption of meat (bovine, poultry, and fish), with the figures significantly higher than in the remaining clusters. The consumption of wheat and vegetables is the lowest of all analyzed clusters. The statistics for eggs are also close to the lowest value of cluster 1.

### Stage II. Cluster Comparisons

The next step of the analysis was to compare the three extracted clusters in terms of digit ratio and demographic values. Fisher *F* test was used for cluster comparisons.

#### Digit Ratio

The clusters were compared in terms of digit ratio (26) – Table 2.

**Table 2:** **Digit ratio values in each cluster – based on Manning et al. (26)**.

		Cluster 1	Cluster 2	Cluster 3
**R2D:4D M**	Mean (SD)	0.9855 (0.0013)	0.9834 (0.0028)	0.9833 (0.0024)
	Fisher’s *F*	1.259 (*p* = 0.300)		
**L2D:4D M**	Mean (SD)	0.9860 (0.0012)	0.9863 (0.0024)	0.9843 (0.0044)
	Fisher’s *F*	3.510 (*p* = 0.045)		
**R2D:4D F**	Mean (SD)	0.9980 (0.0027)	0.9939 (0.0046)	0.9914 (0.0032)
	Fisher’s *F*	4.353 (*p* = 0.023)		
**L2D:4D F**	Mean (SD)	0.9980 (0.0048)	0.9930 (0.0038)	0.9905 (0.0023)
	Fisher’s *F*	7.165 (*p* = 0.003)		
**resR2D:4D**	Mean (SD)	0.0030 (0.0034)	0.0006 (0.004)	−0.0019 (0.003)
	Fisher’s *F*	3.167 (*p* = 0.059)		
**resL2D:4D**	Mean (SD)	0.0050 (0.0057)	−0.0002 (0.0035)	−0.0015 (0.0023)
	Fisher’s *F*	5.111 (*p* = 0.013)		

*Mean values and SD. Fisher F for clusters comparisons*.

The clusters differed in the following parameters: left hand digit ratio in men (L2D:4D M) and women (L2D:4D F), right hand digit ratio in women (R2D:4D F), and residuals from the left hand digit ratio analysis (resL2D:4D). Mean values reported by Manning et al. (26) for the total population of participants from 29 countries were as follows: 2D:4D right hand 0.984 ± 0.003 for men and 0.994 ± 0.004 for women; 2D:4D left hand: 0.985 ± 0.002 for men and 0.993 ± 0.004 for women.

In cluster 1, L2D:4D M values were in the middle for clusters, but still higher than the mean value for men. In women, both L2D:4D and R2D:4D were the highest of all analyzed clusters and higher than the population mean. The resL2D:4D value was positive and higher than in the other clusters.

L2D:4D of men in cluster 2 was the highest and higher than the general population mean compared to the other clusters. L2D:4D F and R2D:4D F values were average relative to the remaining clusters and approximated the population means. The value of resL2D:4D was negative but close to zero, which is the intermediate value out of the three clusters.

In cluster 3, L2D:4D for men was the lowest of all clusters of interest and close to the population mean. In women, both L2D:4D and R2D:4D values were the lowest among the three clusters, lower even than the mean for men. The resL2D:4D value was negative, the lowest of all clusters.

#### Demographic Variables

Since previous studies have indicated that the digit ratio is related to social variables (26, 52, 55), the three clusters were analyzed for differences in terms of such variables (Table 3). Differences between clusters were found for the following variables: Sex ratio in a population (sex ratio); gender parity index (girls to boys) in primary level enrollment (GPI_primary); gender parity index (girls to boys) in secondary level enrollment (GPI_secondary); gender parity index (girls to boys) in tertiary level enrollment (GPI_tertiary); median age in male (Mdn_age_M); median age in female (Mdn_age_F); infant mortality rate (deaths/1,000 live births) in male (IMR_M); infant mortality rate (deaths/1,000 live births) in female (IMR_F); birthrate (births/1,000 population); death rate (deaths/1,000 population); total fertility rate – children born/woman (TFR); military expenditures of GDP% (Military_exp); education expenditures of GDP% (Education_exp); health expenditures of GDP% (Health_exp); alcoholic beverages supply quantity (kilograms per capita per year) (alcohol).

**Table 3:** **Demographic variables in each cluster**.

		Cluster 1	Cluster 2	Cluster 3
**Sex ratio**	Mean (SD)	0.9650 (0.039)	0.9543 (0.022)	0.9827 (0.018)
	Fisher’s *F*	4.552 (*p* = 0.020)		
**GPI_primary**	Mean (SD)	0.9875 (0.013)	0.9957 (0.006)	0.9982 (0.012)
	Fisher’s *F*	1.8327 (*p* = 0.180)		
**GPI_secondary**	Mean (SD)	0.9550 (0.047)	0.9921 (0.031)	1.0282 (0.049)
	Fisher’s *F*	5.3597 (*p* = 0.011)		
**GPI_tertiary**	Mean (SD)	1.1600 (0.29)	1.3021 (0.145)	1.4200 (0.215)
	Fisher’s *F*	2.8431 (*p* = 0.076)		
**Md_age_M**	Mean (SD)	38.32 (6.44)	40.86 (2.07)	37.26 (3.01)
	Fisher’s *F*	3.9597 (*p* = 0.031)		
**Md_age_F**	Mean (SD)	40.4 (7.16)	43.31 (1.45)	39.50 (3.65)
	Fisher’s *F*	3.922 (*p* = 0.032)		
**IMR_M**	Mean (SD)	10.69 (8.58)	5.35 (3.83)	5.1318 (2.26)
	Fisher’s *F*	2.864 (*p* = 0.075)		
**IMR_F**	Mean (SD)	9.10 (7.86)	4.24 (2.43)	4.2118 (1.73)
	Fisher’s *F*	3.646 (*p* = 0.040)		
**Birthrate**	Mean (SD)	10.94 (3.95)	10.01 (1.15)	12.67 (2.21)
	Fisher’s *F*	5.024 (*p* = 0.014)		
**Death rate**	Mean (SD)	9.67 (2.50)	10.53 (1.64)	8.28 (1.48)
	Fisher’s *F*	5.334 (*p* = 0.011)		
**TFR**	Mean (SD)	1.55 (0.39)	1.54 (0.22)	1.88 (0.23)
	Fisher’s *F*	6.141 (*p* = 0.006)		
**Military_exp**	Mean (SD)	1.7525 (0.42)	1.2550 (0.39)	1.5600 (1.12)
	Fisher’s *F*	0.889 (*p* = 0.423)		
**Education_exp**	Mean (SD)	3.9250 (0.70)	5.5714 (1.24)	6.3273 (0.78)
	Fisher’s *F*	7.973 (*p* = 0.002)		
**Health_exp**	Mean (SD)	7.7750 (1.74)	9.5857 (1.81)	10.2273 (2.68)
	Fisher’s *F*	1.860 (*p* = 0.175)		
**Alcohol**	Mean (SD)	61.72 (34.72)	111.50 (26.48)	102.81 (31.76)
	Fisher’s *F*	4.4143 (*p* = 0.022)		

*Mean values and SD. Fisher F for clusters comparisons*.

*Note: abbreviations are explained earlier in the text*.

In terms of demographics, cluster 1 stood out due to very high (almost twice as high as the other clusters) infant mortality rate (difference significant for females), low GPI in secondary level enrollment, and the lowest education expenditures among clusters. In addition, alcoholic beverages supply was the lowest. The other variables are the middle values among the three clusters, with TFR close to the lowest value recorded in cluster 2.

Of note in cluster 2 are the highest median age in male and female of all clusters, the lowest birthrate and highest death rate, the lowest TFR and highest alcoholic beverages supply. Infant mortality rate (difference statistically significant for females) is the average for clusters, although similar to the lowest value in cluster 3.

Cluster 3 has a low (and the lowest of all clusters) infant mortality rate (difference statistically significant for females), the highest birthrate and lowest death rate, as well as the highest TFR (significantly higher than in the other two clusters). Median age in males and females is the lowest of all analyzed clusters. GPI in secondary level enrollment is the highest among clusters and greater than one, meaning that there are more women at this stage of education. Education expenditure is also the highest among the three clusters.

## Discussion

By means of cluster analysis, it was possible to identify three groups of countries that differ in the consumption of basic food products, i.e., meat, wheat, etc. Cluster 1, which includes countries in the Mediterranean and Black Sea region, is characterized by a plant-based diet (wheat and vegetables) and low consumption of animal source foods (all types of meat plus eggs). Cluster 3 includes mainly Scandinavian, Northern European, and large non-European countries (such as Canada, Australia, and USA). Most of them are high-income Western countries, with wide access to sea. The diet in these countries is based on meat (mainly bovine, poultry, and fish), with low consumption of plant products (wheat and vegetables) and eggs. Cluster 2 includes Central and Western European countries. They exhibit high consumption of pig meat and eggs, which constitute the main components of the diet. However, overall meat consumption is considerably lower than in cluster 3.

The next step of the analysis revealed that the clusters differed not only in the diet type but also in terms of morphometric data, in this case, the digit ratio. The differences were seen mostly in left hand 2D:4D, since the right hand only discriminated clusters for women. Detailed analysis of clusters showed that countries with predominantly plant-based diets (cluster 1) have high digit ratio values both in males and females, i.e., low level of prenatal estrogen and/or high level of prenatal testosterone. By contrast, in countries with mainly meat-based diets (cluster 3), the male pattern of digit ratio is dominant in both sexes, suggesting high prenatal estrogen and/or low prenatal testosterone exposure *in utero*. Cluster 2 countries have average parameters for the clusters of interest, with sex-typical digit ratios.

These findings show an important correlation between the type of diet and digit ratio distribution in a population. A protein-rich, meat-based diet appears to be associated with masculine 2D:4D, while plant-based diet with feminine 2D:4D. Since the digit ratio is believed to be set *in utero* through the effects of prenatal sex steroids (29–31), we can conclude that diet and levels of these hormones are indeed related. Specifically, a high-protein diet would be associated with high level of prenatal testosterone (and/or low level of prenatal estrogen), while a plant-based one – with low level of prenatal estrogen (and/or high level of prenatal testosterone). These conclusions are consistent with other findings on the effects of diet on sexual characteristics [e.g., for protein – (21); phytoestrogens – (56, 57)].

In the analysis of the relationship between digit ratio and demographic variables, Manning et al. (26) focused mainly on gender inequality indices. The analysis of differences between cluster discussed in the present paper identified additional variables that may corroborate that relationship.

The most salient figures in cluster 1 are high infant mortality rate, low TFR, the lowest participation of girls in education, and low education expenditures. These indices analyzed in the context of other variables, such as a plant-based diet and the feminine digit ratio add to the picture of this group of countries as less wealthy and underdeveloped relative to other clusters. The opposite demographic data describe cluster 3: the lowest infant mortality rate along with the highest TFR, education expenditures, and proportion of girls in education. The affluence of this group of countries is also reflected in their meat-based diets. These variables are correlated with the masculine digit ratio. Cluster 2 values for these demographic data are in the middle. On the other hand, these countries have the highest median age for both sexes and low TFR, confirming the claim that the population of Central and Western Europe is aging.

## Conclusion

This paper presents preliminary analyses of correlations between population diet profile (a macro-environmental factor) and sexual dimorphism measured by 2D:4D ratio. In countries with high consumption of meat, male-like pattern of digit ratio is observed in both sexes. It may suggest that, in these countries, a meat-based diet is followed by males and females. The same countries appear to be the most developed and wealthy. It must be noted, however, that it is not possible, on the basis of this type of data, to pinpoint the mechanisms underlying these relationships. Especially, that all analyzed variables are interlinked on multiple levels.

The existing data support the presence of the processes running at various levels that have a significant impact on the evolution of contemporary human societies. In the light of the findings confirming the fast pace of evolutionary changes in Homo sapiens (58), the role of diet, as one of the factors affecting the fitness of the human population, should probably be reevaluated from a multidisciplinary and multigenerational perspective.

## Author Contributions

Both authors contributed to study design, data analysis, and manuscript writing.

## Conflict of Interest Statement

The authors declare that the research was conducted in the absence of any commercial or financial relationships that could be construed as a potential conflict of interest. The reviewer KS and handling Editor declared their shared affiliation, and the handling editor states that the process nevertheless met the standards of a fair and objective review.

## Footnotes

^1^http://faostat3.fao.org.

^2^https://www.cia.gov/library/publications/the-world-factbook/.
