# Supplementary material for: Exploratory Analysis of the Links among Food Consumption Profiles, Prenatal Androgens, and Selected Measures of Quality of Life
Source: Front Public Health. 2016 Oct 26;4:240. doi: 10.3389/fpubh.2016.00240 (PMC5080284; doi:10.3389/fpubh.2016.00240)
Supplement: Supplementary file 1 [file data_sheet_1.pdf]

Mean food supply quantity (kg/capita/year) of the years 2000-2011 in selected countries. Source: http://faostat3.fao.org.

	Bovine	Pigmeat	Poultry	Eggs	Milk	Wheat	Stimulants	Fruits	Vegetables	Fish & seafood
**Argentina**	55,53	6,88	27,63	8,97	87,82	107,13	7,99	83,00	68,58	7,34
**Australia**	39,84	21,08	36,74	5,87	100,73	69,33	6,81	99,42	98,08	24,49
**Austria**	18,14	71,65	17,78	13,25	33,57	82,63	9,03	137,78	96,93	12,56
**Belgium**	19,17	35,08	22,78	12,23	61,67	107,63	6,71	76,81	124,58	24,32
**Bulgaria**	7,89	21,81	18,48	10,71	87,25	123,36	5,65	41,21	94,73	4,88
**Canada**	32,20	27,17	36,76	11,47	41,53	85,88	8,10	128,97	118,98	23,41
**Croatia**	7,98	33,21	9,33	10,43	117,08	101,37	6,93	94,63	90,91	15,69
**Czech Republic**	7,83	45,24	24,42	14,83	34,31	100,98	6,57	69,53	74,29	9,78
**Denmark**	27,03	28,60	20,07	17,37	38,35	101,90	13,42	120,28	102,48	22,63
**Finland**	18,50	33,55	17,50	8,63	128,37	79,65	13,78	90,14	77,13	33,60
**France**	26,36	34,53	23,71	14,53	52,70	101,25	9,67	107,46	104,55	33,85
**Germany**	12,25	53,20	15,05	12,17	69,60	81,73	8,93	86,22	89,63	14,38
**Greece**	20,38	30,63	15,68	9,20	86,36	126,47	7,55	154,98	257,89	20,97
**Hungary**	8,13	44,58	30,68	15,63	69,07	104,90	4,45	73,26	107,68	4,93
**Iceland**	12,39	19,76	20,57	8,17	94,48	67,98	13,08	117,59	63,79	90,29
**Ireland**	24,23	36,18	27,20	7,78	158,05	105,42	6,78	119,39	82,08	22,33
**Italy**	23,68	39,77	16,73	11,79	36,93	148,04	7,41	146,83	166,01	24,68
**Netherlands**	17,23	40,03	16,73	16,55	129,53	66,98	8,23	131,38	91,85	21,46
**New Zealand**	27,20	20,48	33,43	10,02	54,81	75,56	4,26	111,64	130,32	26,01
**Norway**	20,29	23,60	13,59	10,34	65,88	101,28	13,09	128,81	73,67	51,60
**Poland**	5,10	49,21	19,47	11,23	35,66	109,13	4,63	51,10	122,37	10,33
**Portugal**	17,17	41,31	26,11	9,66	57,18	97,15	6,43	115,15	176,86	55,65
**Romania**	7,68	28,33	17,91	13,25	14,78	138,63	3,25	63,44	174,70	4,83
**Spain**	14,76	57,33	27,08	14,31	99,00	85,38	6,60	98,57	152,25	42,63
**Sweden**	23,10	36,63	13,83	11,36	73,90	80,22	12,23	113,76	85,06	31,22
**Switzerland**	20,69	33,99	14,56	10,29	101,03	93,11	8,43	88,41	96,74	16,09
**Turkey**	5,94	0,00	13,86	9,33	106,50	195,32	3,71	116,62	242,29	7,67
**United Kingdom**	19,97	25,88	29,68	10,19	118,82	97,64	7,78	117,32	91,32	20,18
**USA**	41,44	29,59	50,58	14,41	120,25	83,58	7,34	110,98	124,54	22,67

Demographic variables in selected countries. Source: https://www.cia.gov/library/publications/the-world-factbook/ and http://faostat3.fao.org.

	Sex Ratio	GPI_prim	GPI_second	GPI_tertiary	Md_age _M	Md_age _F	IMR_M	IMR_F	Birth rate	Death rate	TFR	Military_exp	Education _exp	Health_exp	Alcohol
**Argentina**	0,97	0,99	1,14	1,52	30,10	32,30	11,15	8,71	16,88	7,36	2,29	0,91	6,30	8,10	67,17
**Australia**	1,01	1	0,96	1,32	37,50	39,00	4,74	4,10	12,19	6,94	1,77	1,71	5,60	9,00	109,10
**Austria**	0,95	0,99	0,96	1,18	45,30	43,20	5,01	3,27	8,69	10,23	1,41	0,81	5,90	10,60	144,53
**Belgium**	0,96	1	0,97	1,25	41,70	44,40	4,67	3,66	9,99	10,63	1,65	1,05	6,60	10,60	120,95
**Bulgaria**	0,92	1	0,96	1,32	40,30	44,80	18,07	11,91	8,92	14,32	1,43	1,46	4,10	7,60	79,60
**Canada**	0,99	1	0,98	1,36	40,40	42,90	5,04	4,37	10,29	8,09	1,59	1,24	5,40	11,20	97,08
**Croatia**	0,93	1	1,04	1,27	40,20	43,90	5,99	5,73	9,49	11,99	1,44	1,70	4,30	7,80	113,23
**Czech Republic**	0,95	1	1,01	1,38	39,60	42,30	2,76	2,49	9,79	10,94	1,27	1,08	4,20	7,40	170,72
**Denmark**	0,97	1,01	1,03	1,47	40,70	42,50	4,17	4,02	10,22	10,19	1,74	1,41	8,70	11,20	119,33
**Finland**	0,96	0,99	1,05	1,23	41,20	45,00	3,65	3,06	10,35	10,33	1,73	1,47	6,80	8,90	104,12
**France**	0,96	0,99	1,01	1,28	39,30	42,40	3,63	2,97	12,49	8,85	2,08	1,90	5,90	11,60	88,46
**Germany**	0,97	1	0,95		45,10	47,20	3,75	3,14	8,42	11,04	1,41	1,35	5,10	11,10	137,12
**Greece**	0,96	1	0,95	1,1	42,40	44,60	5,24	4,28	8,80	10,80	1,39	1,72	4,10	9,00	65,26
**Hungary**	0,91	0,99	0,99	1,37	39,10	43,50	5,36	4,81	9,26	12,70	1,41	0,83	4,90	7,70	109,08
**Iceland**	1,00	1	1,03	1,92	35,90	36,90	3,30	3,00	13,90	7,02	1,89	0,13	7,60	9,10	77,40
**Ireland**	1,00	1,01	1,06	1,2	35,40	36,10	4,11	3,35	15,18	6,38	2,10	0,55	6,40	9,40	185,08
**Italy**	0,93	0,99	0,99	1,42	43,30	45,60	3,51	3,10	8,84	9,93	1,40	1,69	4,50	9,50	72,69
**Netherlands**	0,98	0,99	0,98	1,12	41,20	42,90	3,95	3,35	10,83	8,39	1,78	1,27	5,90	12,00	98,93
**New Zealand**	0,99	1,01	1,04	1,45	36,70	38,40	5,14	4,01	13,40	7,20	2,07	1,13	7,40	10,10	87,94
**Norway**	0,98	1	0,98	1,64	38,20	39,90	2,79	2,15	12,09	9,22	1,77	1,40	6,90	9,10	74,02
**Poland**	0,94	0,99	1	1,43	37,90	41,30	6,88	5,45	9,77	10,24	1,31	1,91	5,20	6,70	97,23
**Portugal**	0,95	0,97	1,04	1,19	39,00	43,40	4,92	4,02	9,42	10,86	1,51	1,78	5,60	10,40	115,76
**Romania**	0,95	0,99	0,99	1,34	38,40	41,40	11,52	8,72	9,27	11,84	1,30	1,29	4,20	5,90	95,56
**Spain**	0,97	0,99	1,04	1,24	40,40	42,90	3,66	2,97	9,88	8,88	1,48	0,86	5,00	9,60	110,19
**Sweden**	0,98	0,99	0,99	1,58	40,20	42,20	2,88	2,30	11,92	10,21	1,67	1,18	7,00	9,40	70,52
**Switzerland**	0,97	1	0,96	1,01	41,00	42,90	4,12	3,32	10,48	8,80	1,53	0,76	5,20	10,90	101,13
**Turkey**	1,02	0,97	0,89	0,78	29,20	30,00	22,48	20,32	16,86	6,10	2,13	2,31	2,90	6,70	13,37
**United Kingdom**	0,99	1	1,02	1,39	39,20	41,60	4,86	4,00	12,22	9,33	1,91	2,49	6,20	9,30	113,13
**USA**	0,97	1,01	1,01	1,4	36,30	39,00	6,75	5,56	13,42	8,39	2,06	4,35	5,40	17,90	100,08

Sex Ratio - sex ratio in a population; GPI_prim - Gender Parity Index (girls to boys) in primary level enrolment; GPI_second - Gender Parity Index (girls to boys) in secondary level enrolment; GPI_tertiary - Gender Parity Index (girls to boys) in tertiary level enrolment; Mdn_age_M - Median age in male; Mdn_age_F - Median age in female; IMR_M - Infant mortality rate (deaths/1,000 live births) in male; IMR_F - Infant mortality rate (deaths/1,000 live births) in female; Birth rate (births/1,000 population); Death rate (deaths/1,000 population); TFR - Total fertility rate - children born/woman; Military_exp - Military expenditures of GDP %; Education_exp - Education expenditures of GDP %; Health_exp - Health expenditures of GDP %; Alcohol - Alcoholic beverages supply quantity (kg/capita/year).
